# Estimating intervention effectiveness in trials of malaria interventions with contamination

**DOI:** 10.1186/s12936-021-03924-7

**Published:** 2021-10-20

**Authors:** Lea Multerer, Fiona Vanobberghen, Tracy R. Glass, Alexandra Hiscox, Steven W. Lindsay, Willem Takken, Alfred Tiono, Thomas Smith

**Affiliations:** 1 Swiss Tropical and Public Health Institute, Basel, Switzerland; 2 University of Basel, Basel, Switzerland; 3 Laboratory of Entomology, Wageningen University & Research, Wageningen, The Netherlands; 4 ARCTEC, London School of Hygiene and Tropical Medicine, London, UK; 5 Department of Biosciences, Durham University, Durham, UK; 6 Centre National de Recherche et de Formation sur le Paludisme, Ouagadougou, Burkina Faso

**Keywords:** Stepped wedge cluster randomized trial, Contamination, Sigmoid random effects model, Contamination range, Effective coverage, Malaria

## Abstract

**Background:**

In cluster randomized trials (CRTs) or stepped wedge cluster randomized trials (SWCRTs) of malaria interventions, mosquito movement leads to contamination between trial arms unless buffer zones separate the clusters. Contamination can be accounted for in the analysis, yielding an estimate of the contamination range, the distance over which contamination measurably biases the effectiveness.

**Methods:**

A previously described analysis for CRTs is extended to SWCRTs and estimates of effectiveness are provided as a function of intervention coverage. The methods are applied to two SWCRTs of malaria interventions, the SolarMal trial on the impact of mass trapping of mosquitoes with odor-baited traps and the AvecNet trial on the effect of adding pyriproxyfen to long-lasting insecticidal nets.

**Results:**

For the SolarMal trial, the contamination range was estimated to be 146 m ($95\%$ credible interval $[0.052,\,0.923]$ km), together with a $31.9\%$ ($95\%$ credible interval $[15.3,\,45.8]\%$) reduction of *Plasmodium* infection, compared to the $30.0\%$ reduction estimated without accounting for contamination. The estimated effectiveness had an approximately linear relationship with coverage. For the AvecNet trial, estimated contamination effects were minimal, with insufficient data from the cluster boundary regions to estimate the effectiveness as a function of coverage.

**Conclusions:**

The contamination range in these trials of malaria interventions is much less than the distances *Anopheles* mosquitoes can fly. An appropriate analysis makes buffer zones unnecessary, enabling the design of more cost-efficient trials. Estimation of the contamination range requires information from the cluster boundary regions and trials should be designed to collect this.

## Background

When testing new malaria control interventions, cluster randomized trials (CRTs) are often the study design of choice, because the intervention is either assigned at the household level, or contamination effects are anticipated between the households [1]. With malaria, most transmission happens during the night when *Anopheles* mosquitoes bite and people are in their home. Movement of mosquitoes while searching for human hosts or oviposition sites is therefore the main cause of contamination in trials of mosquito control interventions against malaria. Whereas this is a challenge in field trials, the practical consequence is that intervention has a beneficial community effect on individuals living close by. To prevent this effect from biasing trial estimates of efficacy towards the null, clusters are usually chosen as geographically contiguous areas of households.

Since contamination may still arise at the cluster boundaries and hence bias trial results, malaria trials are often designed by choosing well separated clusters or enforcing separation by defining buffer zones around each cluster [2–7]. Ideally, buffer size might be determined using estimates of the range of the contamination [8], but very broad buffers are often used since other information is rarely available. In such trials, entire clusters receive the intervention, but only data from cluster cores are analyzed. This allows a standard analytical approach [2] but at the cost of enrolling very large populations. Spatial separation may increase heterogeneity between clusters, and the cluster cores may be unrepresentative of the whole population if clusters correspond to natural units such as villages.

Estimating the spatial contamination is of scientific interest [9–13] because protection of people living nearby is an important property of an intervention. Secondary analyses of several CRTs of malaria interventions have estimated contamination effects using linear models with terms measuring the distance between observations from one arm of the study to the nearest observation from the other study arm [10, 14–16]. These analyses all found evidence of spatial effects and tended to demonstrate the importance of accounting for contamination in estimating unbiased effects of the intervention. Nevertheless, these linear models cannot simultaneously provide closed-form estimates of the range over which the contamination is relevant while adjusting the estimate of effectiveness for the contamination. The authors recently demonstrated that this can be achieved with a sigmoid random effects model for the analysis of CRTs of malaria interventions with contamination arising from mosquito movement [9].

Stepped wedge cluster randomized trials (SWCRTs) [2, 17, 18] are a modification of CRTs in which the intervention is introduced progressively to all clusters in random order. To gain a better understanding of the effect of contamination in SWCRTs, the proposed model for CRTs [9] is extended to analyze SWCRTs. It is then shown how the measurable contamination between trial arms (a quantity termed contamination range) leads to an estimate of the effective intervention coverage for each household and how this relates to the intervention effectiveness. These methods are applied to two SWCRTs of malaria interventions; the SolarMal trial assessing the effect of mass trapping with solar-powered odor-baited mosquito traps on Rusinga island, Kenya [19–21], and the AvecNet trial investigating the effect of adding pyriproxyfen to long-lasting insecticidal nets in Burkina Faso [22, 23].

## Methods

### The SolarMal trial of odor-baited mosquito traps

The SolarMal SWCRT aimed to reduce mosquito population size, reduce biting intensity and eliminate *Plasmodium falciparum* malaria on Rusinga island, Lake Victoria, Kenya [19–21] using Solar-powered Mosquito Trapping Systems [24] (SMoTS) which included odor-baited traps to lure and kill host-seeking mosquitoes. All households on Rusinga (area 44 km^2^, mean population 24,879 [21]) were eligible to take part in the trial and were assigned to clusters using a travelling salesman algorithm, resulting in 81 geographically contiguous clusters of 50–51 households. Between June 2013 and May 2015, SMoTS were installed in one cluster per week, with a randomized order of clusters, until universal coverage of 4358 households was achieved [20].

The primary outcome was clinical malaria in individuals of any age, measured as fever plus a positive rapid diagnostic test (RDT) result and monitored through repeated household visits, secondary outcomes were malaria prevalence, measured by RDT, and mosquito densities. Data on malaria prevalence were collected at four-month intervals resulting in five survey rounds during rollout, at $22\%$, $46\%$, $63\%$, $76\%$ and $95\%$ intervention coverage. In each survey, malaria prevalence was recorded in a $10\%$ random sample of households and clusters were excluded from analysis in the week during which the SMoTS were installed. Further details are given in the study protocol [19].

The clinical incidence of malaria episodes was unexpectedly low, hence the focus of the original analysis [21] shifted to the secondary outcome. Malaria prevalence was reported to be $31.4\%$ [$95\%$ confidence intervals (CI) $[27.5,\,35.1]\%$] lower in intervention clusters (prevalence $23.7\%$, 1552/6550 people) than in control clusters (prevalence $34.5\%$, 2002/5795 people). Including random effects for clustering and survey round with generalized linear mixed models, the effectiveness of SMoTS on malaria prevalence was estimated to be $30.0\%$ ($95\%$ CI $[20.9,\,38.0]\%$).

### The AvecNet trial of long-lasting insecticidal nets

The AvecNet trial assessed the effect of adding pyriproxyfen, an insect growth regulator, to long-lasting insecticidal nets (LLINs) in rural Burkina Faso, an area with intense malaria transmission and highly pyrethroid-resistant vectors. A baseline demographic survey enumerated 63,903 individuals living in 93 villages in an area of 1250 km$^2$ [22]. Over a two-year period, during high malaria transmission seasons, LLINs treated with permethrin were incrementally replaced by LLINs treated with permethrin and pyriproxyfen in a SWCRT with 40 clusters [22, 23], with an overall $95\%$ coverage of nets. Clusters were based on administrative units and an average of 50 children (aged 6–60 months) were selected in each cluster and followed up by passive case detection for clinical malaria at health centers. Each month from June to September in 2014 and 2015, five clusters switched from control to intervention arm.

The primary outcome was clinical malaria, measured as fever plus a positive RDT result for *Plasmodium falciparum*. The child-years at risk and the incidence rate ratio (IRR), were calculated for each month in each group. Data were collected between June to December 2014 and May to December 2015, resulting in nine months with data from both the intervention and control arms. In these 9 months, the mean intervention coverage was 17, 33, 44, 50, 51, 51, 56, 64 and $81\%$. Further details of trial design can be found in the study protocol [22].

The original analysis [23] estimated clinical malaria incidence of 2.0 episodes per child-year in the control group and 1.5 episodes per child-year in the intervention group (IRR 0.88, $95\%$ CI $[0.77,\,0.99]$, estimated from a Poisson model with offset for log-transformed exposure years, a random effect for cluster and fixed effects for month and health facility).

### Analysis of SWCRTs allowing for contamination

**Fig. 1:**
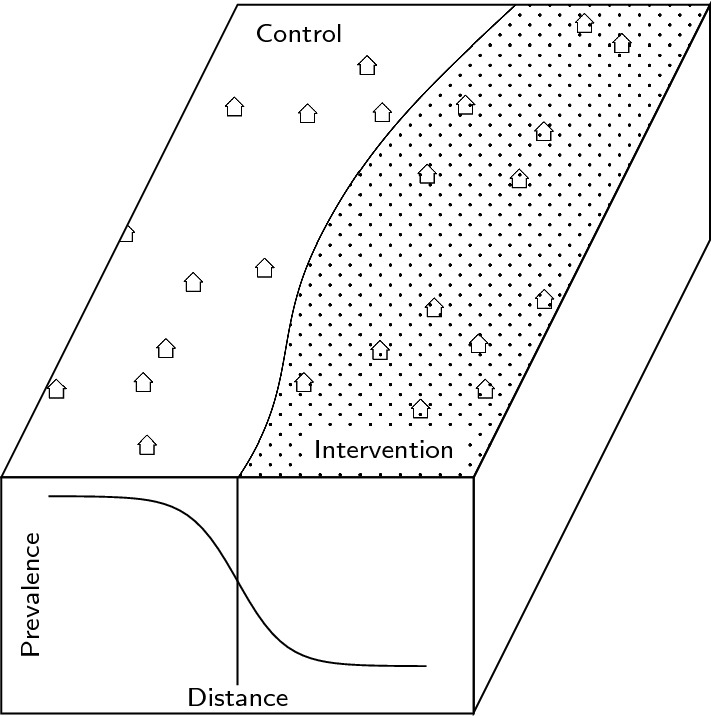
Schematic description of the effect of mosquito movement and the arising contamination in the boundary region of a CRT or SWCRT. On the front face of the rhomboid, the smooth decrease in prevalence between a control and intervention cluster (on the surface) based on the distance of a household to its nearest discordant household is visualized

Contamination between clusters because of mosquito movement between households might be expected to bias the intervention effects in both the SolarMal and AvecNet trials. This contamination is expected to follow a symmetrical smooth gradient in the boundary regions between intervention and control clusters and can hence be modeled by a sigmoid function of the distance of households to the nearest household in the discordant trial arm [9] (Fig. 1). Analyses of simulated datasets [9] found that this approach can provide unbiased and precise estimates of the contamination range and of the effectiveness, given that at least $50\%$ of the households are at distances greater than the estimated contamination range from the nearest discordant household, hereafter called households in core.

With SWCRT designs, while the cluster size is constant, the assignment to arms changes, leading to variation in the distance to the nearest discordant household throughout the study. By accounting for this, and including a random effect for time, it is possible to extend the proposed sigmoid analysis to SWCRTs. Data with all clusters assigned to the intervention or control arm cannot be included, since the distance to the nearest discordant household is then not defined. Let $\Delta _{ijk}$ denote the distance of the *j*th household in the *i*th cluster at the *k*th time step to the nearest discordant household, endowed with a negative sign for the households in the control arm. For malaria prevalence, the outcome $Y_{ijk}$ of the *j*th household in the *i*th cluster at the *k*th time step, with $i = 1,\dots ,c$, $j = 1,\dots ,h$ and $k = 1,\dots ,s$ (hereafter abbreviated with household *ijk*), can then be described by a Bayesian hierarchical model as follows [9]:

1 $$\begin{aligned} Y_{ijk} &\sim {\text {Binomial}} (p_{ijk}),\\ {\text{logit}} (p_{ijk})&= \beta_{1,ik} +{ \frac{\beta _2}{1+\exp (-\beta_3 \Delta _{ijk})}},\\ \beta_{1,ik}&\sim {\text{Normal}} (\mu ,\tau ). \end{aligned}$$

For malaria incidence, a log link function together with an offset for the time at risk should be used. In this model formulation, $\beta _{1,ik}$ denotes a random effect parameter assigned to each cluster in each survey round [2], centered on the expected prevalence in the control arm, $\beta _1$. The other parameters $\beta _2$, $\beta _3$, $\mu$ and $\tau$ are assigned non-informative priors. The parameter $\beta _2$ denotes the intervention effect and $\beta _3$ can be transformed into the contamination range in km as $\hat{\theta } = \log (0.95/0.05)\beta _3^{-1}$.

**Fig. 2:**
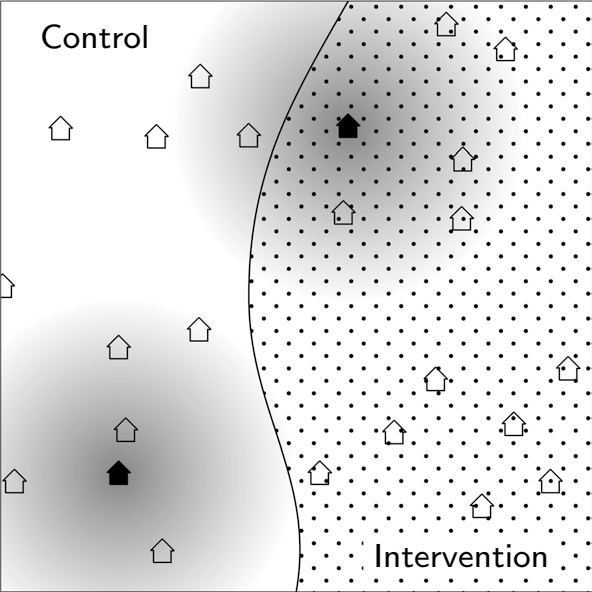
Schematic description of the effective coverage. For two households, one in the intervention arm (upper right corner) and one in the control arm (lower left corner), the area from which the effective coverage is calculated is shaded in grey. The closer a household is to one of these two households, the bigger its impact on the effective coverage (darker shade of grey). For the household in the control arm, the effective coverage is close to zero, since no intervention households are close. For the household in the intervention arm, the effective coverage is more than $50\%$

This estimate $\hat{\theta }$ can be used to define the area around household *ijk* that influences the density of infectious mosquitoes, the effective intervention coverage $\mathcal {R}_{ijk}$. This is defined as the common density of the intervened households relative to the general density of households. The closer any other household *m* is to *ijk*, the greater is the contribution of *m*’s intervention status to the effective intervention coverage (Fig. 2). This leads to a simple relationship between $\mathcal {R}_{ijk}$ and the distance to the nearest discordant household $\Delta _{ijk}$. Households whose distance to the nearest discordant household is large are only surrounded by households with the same intervention status and hence $\mathcal {R}_{ijk}$ is either almost zero or one. By approximating this relationship with a sigmoid function whose growth rate depends on $\hat{\theta }$, and plugging it into the Bayesian hierarchical model 1, it holds that:

$$\begin{aligned} \text {logit}(p_{ijk}) \approx \beta _1 + \beta _2\mathcal {R}_{ijk}. \end{aligned}$$

With this formulation and after a back transformation, it is possible to describe the trial outcome as a linear function of the effective coverage, $\mathcal {R}_{ijk}$. This procedure is formally described in Appendix.

### Analysis of the SolarMal and the AvecNet trials

Both SWCRTs were analyzed with the sigmoid random effects model from Eq. 1 and fitted using rjags [25]. Uninformative priors were chosen for all parameters, and $\beta _3$ was constrained for the contamination range to be positive. All calculations were performed at sciCORE scientific computing core facility at the University of Basel under R version 4.0.0 [26].

For the SolarMal trial, the random effects parameter $\beta _{1,ik}$ was varied by survey round (as in the original publication [21]) and by survey round and households. The results are reported in terms of the reduction in odds ratio (OR), as well as the reduction in relative risk (RR). For the AvecNet trial, the random effects parameter was varied by survey round and health facility and the results are reported in terms of the IRR. Instead of the distance to the nearest discordant household, the distance to the household of the nearest discordant child enrolled in the trial was calculated, because only this data was available. The intervention effectiveness is described in terms of the effective intervention coverage for both trials.

## Results

### SolarMal trial

**Fig. 3:**
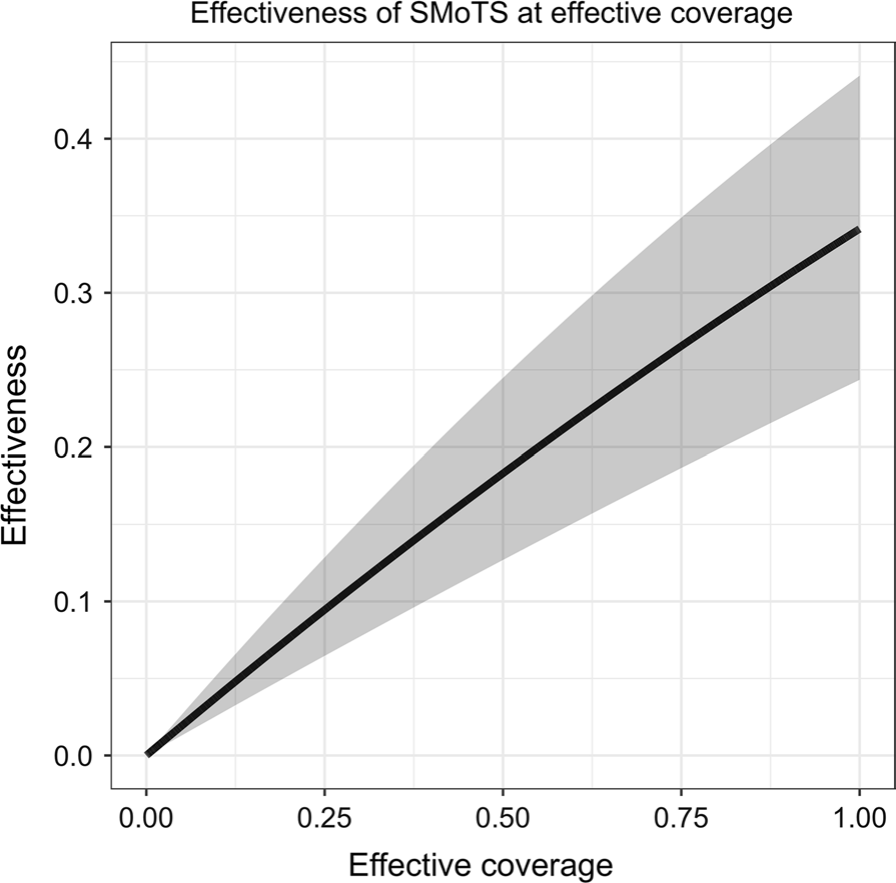
The effectiveness of Solar-powered Mosquito Trapping Systems (SMoTS) (on the *y*-axis) in terms of the effective coverage is visualized in black, with credible intervals in grey. The effectiveness was estimated with the model including a random effect for the household effects (Sigmoid RE + hh)

For the five survey rounds, the mean distance to the nearest discordant household was 2.3, 0.9, 1.1, 1.6 and 2.7 km. For an assumed contamination range of 100 m, this results in 95, 91, 85, 93, and $98\%$ of households in core, justifying a sigmoid random effects analysis [9]. When including a random effect for survey round only, SMoTS were associated with a $31.9\%$ reduction ($95\%$ credible interval (CrI): $[15.3,\,45.8]\%$) in odds ratio in the two arms, translating to a $25.2\%$ reduction ($95\%$CrI: $[10.9,\,39.0]\%$) in relative risk (Table 1). The credible intervals were wider than the original confidence intervals. The contamination range was estimated to be 146 m, also with a wide credible interval ($[0.052,\,0.923]$ km). With another random effect included for the household effects, SMoTS were associated with a $42.1\%$ reduction ($95\%$CrI: $[32.2,\,51.3]\%$) in odds ratio, and a $34.1\%$ reduction ($95\%$CrI: $[24.4,\,44.1]\%$) in relative risk. The contamination range was estimated to be 133 m ($[0.052,\,0.943]$ km). The effectiveness is almost linear in effective coverage (Fig. 3) rising from zero effectiveness at zero coverage to the maximal effectiveness of $34.1\%$ when intervention households are only surrounded by other intervention households. The credible intervals increase with coverage.

**Table 1:** Results for the sigmoid random effects model (Sigmoid RE) and the sigmoid random effects model including a random effect for the households (Sigmoid RE + hh) for the SolarMal trial, compared to a generalized linear mixed effects model from the original analysis [21] (GLMM)

	Sigmoid RE	Sigmoid RE + hh	GLMM [21]
$1-$OR (in $\%$)	31.9, $[15.3,\,45.8]$	42.1, $[32.2,\,51.3]$	30.0, $[20.9,\,38.0]$
$1-$RR (in $\%$)	25.2, $[10.9,\,39.0]$	34.1, $[24.4,\,44.1]$	–
$\hat{\theta }$ (in km)	0.146, $[0.052,\,0.923]$	0.133, $[0.052,\,0.943]$	–

The results are reported as the reduction in odds ratio $1-$OR as well as the reduction in relative risk $1-$RR. The contamination range $\hat{\theta }$ is only estimable for the two sigmoid random effects models

### AvecNet trial

The mean distance to the household of the nearest discordant child enrolled was high for all nine survey rounds, with a mean of 4.5 km. For an assumed contamination range of 100 m, this resulted in a mean of $98\%$ of households in core. This indicates that the data to estimate the contamination range are sparse, but a sigmoid random effects analysis can be carried out.

**Fig. 4:**
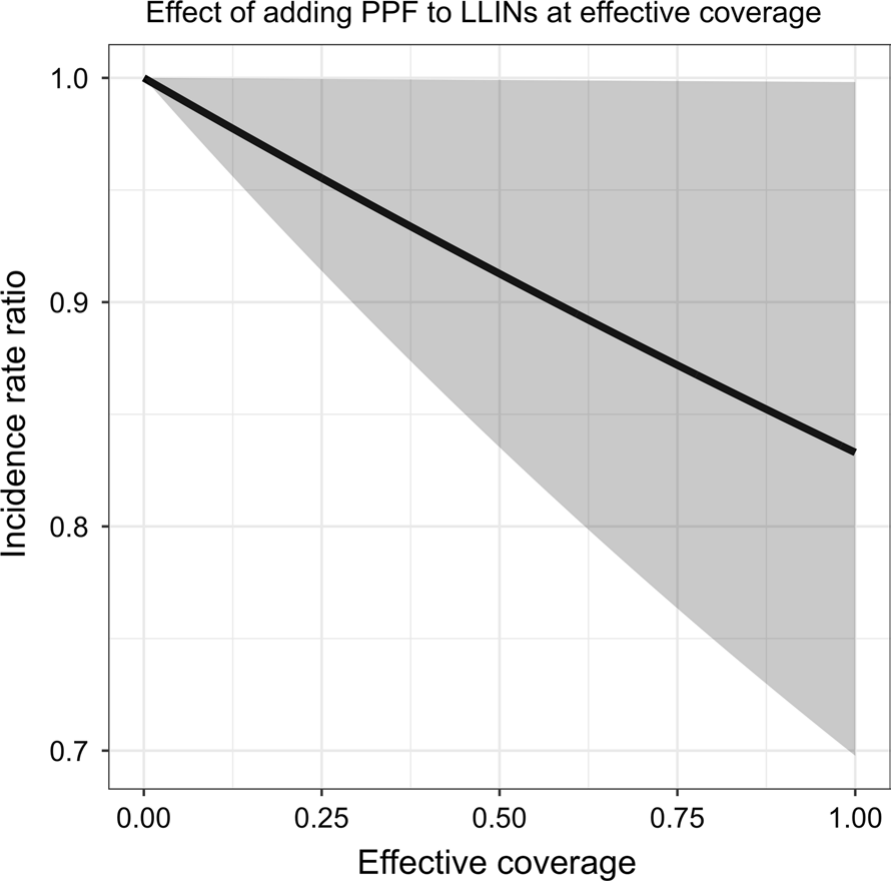
The effect of adding pyriproxyfen (PPF) to long-lasting insecticidal nets (LLINs) (on the *y*-axis) in terms of the effective coverage is visualized in black, with credible intervals in grey

Adding pyriproxyfen to LLINs was associated with a reduction in incidence of clinical malaria in children of $17\%$ (IRR 0.83, $95\%$CrI: $[0.70,\,1.00]$), with credible intervals comparable to the confidence intervals from the original analysis (Table 2). The contamination range was estimated to be 101 m, with a wide credible interval ($95\%$CrI: $[0.051,\,0.745]$ km). The incidence rate ratio decreases as the effective coverage increases, in an almost linear fashion because of the inverse logarithmic transform (Fig. 4). The credible intervals become wider with higher the coverage.

**Table 2:** Results for the sigmoid random effects model (Sigmoid RE) for the AvecNet trial, compared to a generalized linear mixed effects model from the original analysis [23] (GLMM)

	Sigmoid RE	GLMM [23]
IRR	0.83, $[0.70,\,1.00]$	0.88, $[0.77,\,0.99]$
$\hat{\theta }$ (in km)	0.101, $[0.051,\,0.745]$	–

The results are reported in terms of the incidence rate ratio (IRR). The contamination range $\hat{\theta }$ is only estimable for the sigmoid random effects model

## Discussion

In CRTs or SWCRTs of malaria interventions, contamination between the trial arms arises because of mosquito movement. In a conventional analysis this may bias effectiveness estimates, but this can be corrected with an appropriate analysis, such as a Bayesian hierarchical model with a sigmoid function for effectiveness as a function of distance to the nearest discordant household, that was recently proposed for CRTs [9]. This model yields a closed-form contamination range that quantifies the contamination arising from mosquito movement between trial arms, and adjusts the main estimate of effectiveness for contamination, eliminating the need for buffer zones.

The proposed analytical approach is tailor-made for malaria interventions where transmission can be geolocated to the host’s primary residence, and the main source of contamination between clusters arises from dispersal of adult female *Anopheles* mosquitoes, for which the proposed model, corresponding to mosquito dispersion by diffusion [27, 28] is a reasonable approximation. In nature dispersal will vary between sites, within sites and by season, and depends on the extent and spatial distribution of aquatic habitats, households and alternative blood sources [29], as well as wind strength and direction and obstacles in the environment. For both interventions considered here, with effects mainly depending on mosquito densities, contamination was considered to be symmetrical. The intervention may protect nearby nonusers, while users with many nearby nonusers have reduced intervention effects. With this model, a difference between homogeneously distributed and clustered interventions on the overall intervention effect is not distinguishable [29]. The same modelling approach might be applied where the intervention itself is designed to be dispersed by mosquitoes (for instance sterile insect techniques) and even with human-side interventions such as mass drug administration or mass vaccination, though in the latter cases contamination is less important relative to the overall efficacy since more of the impact is due to the direct effect of individual protection. Contamination also arises in CRTs of many other health interventions, but where transmission is not by night-biting mosquitoes the geometry is likely to be more complicated. For instance, where the intervention is behavioural and the primary source of contamination is social (and hence non-spatial), or with directly transmitted infections or those transmitted by less mobile and day biting *Aedes* mosquitoes (where infections often acquired at workplaces or schools, making geographically congruent clusters less desirable), different models of contamination are needed. In any given trial, the appropriateness and fit of the chosen contamination model should be carefully evaluated.

In this work, the sigmoid model is applied to two SWCRTs, the SolarMal and AvecNet trials. SWCRTs can be inferior in terms of power or bias compared to parallel designs and might be vulnerable to imprecision caused by temporal trends in underlying disease rates [3, 30] but may be required because of logistical, practical or financial constraints [1, 31] (for example, in the SolarMal trial an objective was to assess whether interruption of transmission would occur at complete coverage [20]). Because of the changing boundaries, the analysis of contamination effects in SWCRTs is more complicated, but in principle SWCRT data could be used to analyze changing patterns of contamination in time and place. At the same time, it is unclear how the imbalance between arms affects the precision and bias of the resulting estimates.

A reanalysis of the SolarMal trial yielded a slightly higher estimate of effectiveness than was reported in the original trial analysis [21], but with less precision. Adding a random effect for the households increased the estimate of effectiveness with reasonably wide credible intervals. Also for the AvecNet trial, the reduction in incidence of clinical malaria in children was higher than in the original analysis [23], with only slightly less precision. The contamination range was consistently around 140 m in the SolarMal trial and around 100 m in the AvecNet trial, which is much less than the maximal distance *Anopheles* mosquitoes can fly [8, 32].

The SolarMal trial was conducted in a small, densely populated area and had many small clusters. The AvecNet trial, in contrast, was conducted in a much larger area, with a population density 10 times lower than that in the SolarMal trial (around 50 people per km$^2$ compared to more than 500 people per km$^2$). The settlement patterns where these trials were conducted are also different: in the region where the SolarMal trial took place around Lake Victoria, households are scattered, while the area where the AvecNet trial was conducted has villages with tight aggregations of houses, typical of the West African Sahel. These factors affect the percentage of households in core, the percentage of households unaffected by the contamination across cluster boundaries, where a balance is needed for the proposed analysis to yield unbiased and precise estimates. In the AvecNet trial, a subset of children was chosen from each village, to allow for clusters to be chosen as administrative units. This resulted in a high percentage of households in core, though this number is not comparable to the SolarMal trial, because only the distance to the household of the nearest discordant child was calculated. Informed by a previous simulation study [9], it is assumed that with so little information from the boundary regions, the contamination range cannot be estimated reliably and the proposed model is not working.

Like AvecNet, many trials define clusters based on administrative units with cluster boundaries passing through uninhabited areas. However, for contamination effects to be estimable, the trial must be designed to collect information from the boundary zones where contamination is likely. If cluster boundaries can pass through inhabited areas, as in the SolarMal trial, equal-population clusters can be assigned giving a more balanced design with optimal power, therefore requiring fewer participants. When there is contamination there is also empirical information about every level of local coverage from within either a CRT or SWCRT, even without universal overall coverage. This enables extension of the analysis using kernel density estimation to infer from the contamination range how effectiveness depends on intervention coverage. These estimates could be used to support allocation decisions when interventions are deployed, but where resource constraints mean universal coverage is not achievable.

## Conclusions

It was shown how trials with anticipated contamination effects arising from mosquito movement can be analyzed to give unbiased and precise estimates of effectiveness. Guidance is now needed on how to plan trials with adequate power and precision to allow for contamination. Without the need for buffer zones, or for clusters to correspond to villages, cluster size can be reduced to a minimum determined by operational factors or contamination effects, reducing the required numbers of participants in field trials of malaria interventions. This should lead to more cost-efficient trials and a better understanding of the indirect effects of interventions in protecting nearby nonusers.

## Figures and Tables

**Fig. 5:**
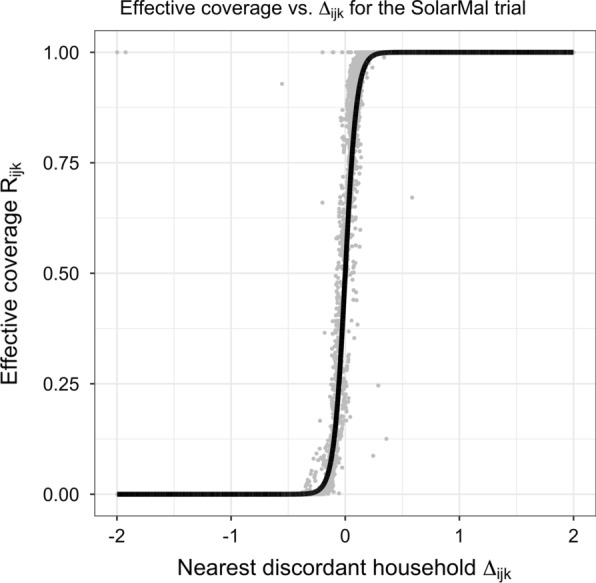
Illustration of the sigmoid relationship between the nearest discordant household and the effective coverage for the SolarMal trial in grey. In black, the sigmoid curve for $\hat{\mathcal {R}}_{ijk}$ that approximates the effective coverage is illustrated. To increase the readability, only households within 2 km of the nearest discordant household are plotted on the *x*-axis

## Data Availability

The data underlying this article will be shared on reasonable request to the corresponding author.

## Appendix

### Estimation of effectiveness as a function of intervention coverage

#### Effective intervention coverage

Assume that the *N* households in the study are ordered such that the first $N_1$ receive the intervention. This means that $(x_m,y_m)$, $m = 1, \dots , N_1$, denote all the coordinates of the $N_1$ intervened households and $(x_m,y_m)$, $m = N_1 + 1, \dots , N$, the coordinates of the control households. For any household with coordinates (*x*, *y*) the effective intervention coverage $\mathcal {R}(x,y)$ is then defined as:

$$\begin{aligned} \mathcal {R}(x,y) = \frac{\sum _{m = 1}^{N_1} K(x-x_m,y-y_m)}{\sum _{n = 1}^{N} K(x-x_n,y-y_n)}, \end{aligned}$$

where *K*(*x*, *y*) is an appropriate function.

If *K*(*x*, *y*) is chosen as 1 whenever $x^2 + y^2 \leqslant \hat{\theta }$, the expression $\mathcal {R}(x,y)$ compares the number of intervened households to the total number of households within the contamination range $\hat{\theta }$ and hence describes the percentage of households within an estimated contamination range $\hat{\theta }$ that receive the intervention, as it has been previously defined [16, 20]. This discrete measurement is imprecise, since it does not consider the closeness between households within the contamination range. Instead, let *K*(*x*, *y*) be a radially symmetric probability density function, for instance a two-dimensional Gaussian kernel. The expression $\mathcal {R}(x,y)$ then represents the common density of the intervened households relative to the general density of households and can be interpreted as a spatial relative risk function, as used in kernel density estimation [33–35].

#### Choice of bandwidth

The bandwidth $\varepsilon$ of the two-dimensional Gaussian kernel

$$\begin{aligned} K(x,y) = \frac{1}{2\pi \varepsilon ^2}\exp \Big (-\frac{x^2 + y^2}{2\varepsilon ^2}\Big ) \end{aligned}$$

is chosen such that $95\%$ of its distribution lies within a radius of the contamination range $\hat{\theta }$ around each household. Taking

$$\begin{aligned} \varepsilon = \frac{\pi }{\sqrt{6}\beta _3} (= 0.44\hat{\theta }) \end{aligned}$$

results in a bivariate normal distribution with $95\%$ of the distribution laying within the contamination range $\hat{\theta }$. This is because the contamination range $\hat{\theta }$ was calculated from $\beta _3$, the growth rate of a sigmoid function. This sigmoid function is the cumulative density function of the one-dimensional logistic distribution with variance $\pi ^2/(3\beta _3^2)$. On the other side, a covariance matrix of a two-dimensional Gaussian kernel gives rise to a variance in one direction quantified by $2\varepsilon ^2$ [9]. The effective intervention coverage of the *i*th household in the *j*th cluster in the *k*th survey round with coordinates $(x_{ijk},y_{ijk})$ can then be calculated as $\mathcal {R}_{ijk} = \mathcal {R}(x_{ijk},y_{ijk})$ and is defined analogously for CRTs, with less indexing.

#### Approximation of the effective intervention coverage

The effective intervention coverage $\mathcal {R}_{ijk}$ can be approximated by

$$\begin{aligned} \hat{\mathcal {R}}_{ijk} = \frac{1}{1 + \exp (-\beta _3\Delta _{ijk})}, \end{aligned}$$

with the same $\beta _3$ as was fitted in the Bayesian hierarchical model

2 $$\begin{aligned} Y_{ijk}&\sim {\text{Binomial}}(p_{ijk}),\\ {\text{logit}}(p_{ijk})&= \beta _{1,ik} + \frac{\beta _2}{1+\exp (-\beta _3\Delta _{ijk})}, \\ \beta _{1,ik}&\sim {\text{Normal}}(\mu ,\tau ). \end{aligned}$$

This relationship between the nearest discordant household $\Delta _{ijk}$ and $\mathcal {R}_{ijk}$ is illustrated in Fig. 5 for the SolarMal trial. The percentage error

$$\begin{aligned} \text {perc. err.} = 100\%\times \frac{\Vert R_{ijk} - \hat{R}_{ijk}\Vert _2}{\Vert R_{ijk}\Vert _2}, \end{aligned}$$

between the effective intervention coverage $\mathcal {R}_{ijk}$ and its approximation $\hat{\mathcal {R}}_{ijk}$ was estimated to be $6\%$ for the SolarMal trial and $3\%$ for the AvecNet trial. These small errors justify this approximation.

#### Estimation of the intervention effectiveness at the effective coverage

Plugging in the inverse relationship between $\Delta _{ijk}$ and $\mathcal {R}_{ijk} \approx \hat{\mathcal {R}}_{ijk}$ in Eq. 2 it holds that

$$\begin{aligned} \text {logit}(p_{ijk}) \approx \beta _1 + \beta _2\mathcal {R}_{ijk} \end{aligned}$$

and the trial outcome can be described as a function of the effective intervention coverage.

## Abbreviations

CRTs: Cluster randomized trials
SWCRTs: Stepped wedge cluster randomized trials
SMoTS: Solar-powered mosquito trapping systems
CI: Confidence intervals
LLIN: Long-lasting insecticidal net
RDT: Rapid diagnostic test
IRR: Incidence rate ratio
OR: Odds ratio
RR: Relative risk
